# In This Issue

**DOI:** 10.1002/cfg.210

**Published:** 2002-10


**Comparative and Functional Genomics**
*Comp Funct Genom* 2002; **3**: 397.
Published online in Wiley InterScience (www.interscience.wiley.com). **DOI:** 10.1002/cfg.210

### In this issue

### Upstream — news in genomics

In this issue we report on important papers published from May to July, highlighting breakthroughs on several important genomes, including mouse, zebrafish, *Fugu* and *Plasmodium*. Other recent papers have reported on a mechanism for genome size reduction in *Arabidopsis*, comparisons and verifications of large-scale protein-protein interaction datasets, developments in RNA interference approaches for mammalian systems and a solid-phase peptide tagging method for proteomics.

### A mathematical analysis of suppression subtraction hybridisation

Chetan Gadgil *et al.* present a mathematical model of SSH based on DNA hybridization kinetics. They have used their model to assess the effects of various experimental parameters (such as mRNA abundance in the cDNA sample and the reaction time used) on the chances of detecting messages from different abundance classes, and on the number of false positives obtained, to facilitate optimisation of the approach. They also use the model to look at the best way to spike a sequence into the driver cDNA pool.

### A computational strategy for protein function assignment that deals with the domain problem

Antonio Perez and colleagues describe and demonstrate an automated approach to protein function classification that combines the keyword (KW) and feature (FT) fields of SWISS-PROT entries to build up a database of protomotifs. When a novel protein shows similarity to an annotated protein, the functional information from a protomotif is only transferred to the novel protein if the match is in the correct region of the protein.

### Meeting Review: ESF — Impact of nucleic acid chemistry on gene function analysis

Kathrin Heermeier *et al.* report on the ESF programme on Integrated Approaches for Functional Genomics workshop "Impact of Nucleic Acid Chemistry on Gene Function Analysis: Antisense, Aptamers, Ribozymes and RNAi".

### Meeting Review: From genotype to phenotype: Linking Bioinformatics and Medical Informatics Ontologies

In first of two reports from Manchester Bioinformatics Week, Ele Holloway reviews the ontologies workshop that launched the conference. The talks covered issues in nomenclature and data standards and profiled a range of existing ontologies and projects currently in development.

### Meeting Review: CCP11 Group Meeting: Towards the Functional Analysis of Microarrays

Clare Sansom reports on the CCP11 group microarray analysis meeting from Manchester Bioinformatics Week. The meeting focussed mainly on statistical methods and analysis, with some presentations on modelling of networks, and profiles of a selection of microarray projects.

### Meeting Review: The 50$^{th}$ American Society for Mass Spectrometry meeting

Francesco Brancia brings us a report from the annual meeting of the ASMS: 'Mass Spectrometry and Allied Topics'. The development of new mass spectrometers and implementation of new analytical methods were the central themes of the conference, and, as last year, the application of mass spectrometry to proteomics was a hot topic.

Copyright © 2002 John Wiley & Sons, Ltd.

